# Influence of hydrogenation on the mechanical properties of Pd nanoparticles†

**DOI:** 10.1039/d0ra08974e

**Published:** 2021-01-13

**Authors:** Jianjun Bian, Liang Yang, Weike Yuan, Gangfeng Wang

**Affiliations:** Department of Engineering Mechanics, SVL, Xi'an Jiaotong University Xi'an 710049 P. R. China wanggf@xjtu.edu.cn; School of Materials Engineering, Jiangsu University of Technology Changzhou 213001 P. R. China

## Abstract

Atomic simulations are conducted to investigate the influence of hydrogenation on the mechanical properties of Pd nanoparticles. It is found that with an increase in the H atom content both the elastic modulus and the yield stress decrease approximately linearly. Moreover, the H atom content evidently alters the atomic deformation mechanisms in Pd nanoparticles. When the H atom content is in the range of 0–0.3, yield initiates from dislocation nucleating beneath surface steps and then a pyramid hillock is formed. Subsequently, dislocation nucleation and exhaustion at the surface will govern the plastic deformation. However, when the H atom content is in the range of 0.3–0.4, massive initial defects are introduced by hydrogenation, which partially suppress the dislocation nucleation around the surface steps, and no pyramid dislocation hillock is formed. Dislocation multiplication will dominate the subsequent plastic deformation. Moreover, as the H atom content increases to 0.4–0.5, the recoverable phase transition plays a key role in the plastic deformation. This study enriches our understanding of the impact of hydrogenation on the mechanical properties and deformation mechanisms of Pd nanoparticles.

## Abstract 2

Atomic simulations are conducted to investigate the influence of hydrogenation on the mechanical properties of Pd nanoparticles.

## Graphical abstract

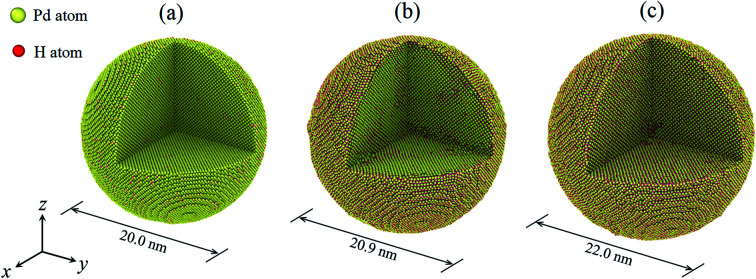

## 1. Introduction

Metallic nanoparticles exhibit a great potential towards applications in such fields as energetic storage, antimicrobial materials, and high-performance catalysts. To fulfill the requirements in practical applications, it is of critical importance to properly describe and understand the mechanical properties and behaviors of these tiny crystalline objects. Owing to the development of experimental and computational technologies, rich types of deformation features of the metallic nanoparticles have been revealed. Based on molecular dynamics (MD) simulations, it was found that gold nanoparticles have size-dependent modulus and yielding stress.^1^ Also, nanoindentation experiments have been performed on gold nanoparticles, and the results revealed that both the modulus and yielding stress of gold nanoparticles are higher than those of the bulk counterparts.^2^ The yielding stress of metallic nanoparticles depends on not only the radius but also the surface morphologies, such as the surface steps and amorphous surface layers.^3,4^ Based on the *in situ* high resolution transmission electron microscopy (HTEM) results, it was revealed that silver nanoparticles with a radius of sub-10 nm could deform like a liquid droplet at room temperature.^5^

The plastic deformation behaviors of metallic nanoparticles also exhibit exceptional characteristics. Surface steps severely influence the incipient plastic deformation of nanoparticles. For some fcc nanoparticles under [001] compression, initial dislocations tend to nucleate at the fringes of surface steps and then form typical pyramid dislocation hillocks beneath the contact area.^6,7^ In nanoindentation experiments, the reversible dislocation associated plasticity has been explicitly observed for silver nanoparticles,^8^ which also plays a dominant role in the adhesive contact between gold polyhedral nanoparticles.^9^ Typically, low dimensional nanostructures prefer to stay in a ‘dislocation starved’ state even after severe deformation. However, in spherical bcc iron nanoparticles, dislocation slip and deformation twinning prevail in the overall plasticity,^10^ and the cross-split of edge dislocations in faceted iron nanoparticles contributes to the strain burst under compression.^11^ The plastic behaviors in the severe deformation of nanoparticles could be tuned by introducing some internal defects as twin boundaries. For example, both the strength and malleability of the twinned metallic nanoparticles are greatly enhanced compared to those of the twin-free samples.^12^ In addition, the flow stress of nanoparticles is also significantly affected by the amorphous surface layer,^4^ where the serrated stress fluctuation is prohibited due to the existence of the amorphous surface layer.

As a type of typical fcc nanoparticles, Pd nanoparticles are widely used for hydrogen storage.^13^ The mechanical properties of Pd nanoparticles are keys in such applications. It was revealed that metals could be deteriorated drastically (even if only a little amount of hydrogen is dissolved), leading to severe structure failures such as embrittlement, crack propagation, and corrosion.^14,15^ Under a high environmental gas pressure, hydrogen atoms could be continuously pumped into the palladium lattice.^16^ After hydrogenation, H atoms occupy the interstitial sites of the Pd lattice, resulting in the expansion of the Pd lattice and an increase in the dislocation density. By using a diffusive molecular dynamics (DMD) model, it is revealed that as H atoms are absorbed by Pd nanoparticles misfit dislocation emerges over the interface between different hydride phases.^17^ This study mainly focuses on the H atom absorption dynamics. However, the influence of hydrogenation on the mechanical properties of Pd nanoparticles has not been fully understood. In the present study, a series of MD simulations are performed to investigate how hydrogenation influences the mechanical properties of Pd nanoparticles, aiming to provide some guidance for designing hydrogen storage materials.

## 2. Simulation methodology

To study the mechanical properties of the hydrogenated Pd nanoparticles, uniaxial compression is conducted along the [001] orientation *via* MD simulations. The larger-scale open-source simulator, LAMMPS, is employed for all the simulations.^18^ The atomic interactions of the Pd–H atomic system are described by the embedded-atom method (EAM).^19^ Based on EAM, the total cohesive energy *E* of a system with *N* atoms is given as1
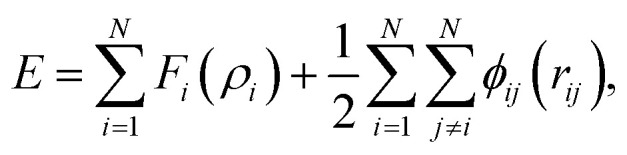
where *F*_*i*_ is the embedding energy of the *i*-th atom, and *ρ*_*i*_ is the atomic electron density with respect to the position of the *i*-th atom. *ϕ*_*ij*_ is the pair-wise interaction energy, which is a function of the atomic separation of the atom pairs *i* and *j*. For the hydrogenated Pd nanoparticles, a potential function file parameterized by Zhou *et al.* is adopted here,^20^ which successfully predicts such parameters of Pd–H atomic systems as lattice constants, cohesive energies, elastic constants of Pd, H, and PdH_*x*_ phases of various compositions.

**Fig. 1:**
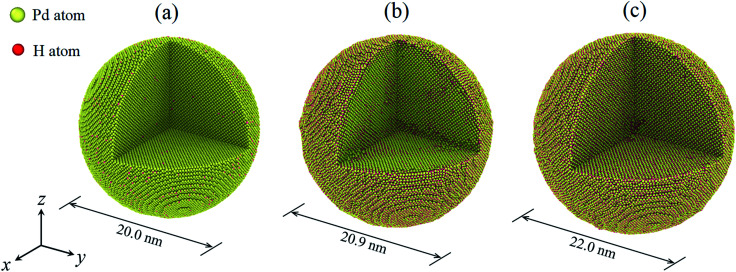
The hydrogenated Pd nanoparticles with different H atom contents: (a) 0.01, (b) 0.33, (c) 0.50. The lattice orientations along *x*-, *y*- and *z*-directions are [100], [010] and [001], respectively. One eighth of the nanoparticle is removed in order to display the H atom distribution on the cross section.

In the present study, the H atom content of a hydrogenated nanoparticle is defined as the ratio of the H atom number to the total atom number of the nanoparticle. To construct a hydrogenated Pd nanoparticle, a defect-free single crystal bulk Pd is first created with the fcc lattice, with a lattice constant of 3.859 Å. Then, according to a specified H atom content, H atoms are randomly added to the octahedral sites of the fcc lattice. Nanoparticles are then carved out of the Pd crystal using a spherical cutting surface with a radius of 10 nm. In this way, the number of Pd atoms is identical in all the nanoparticles and only the H atom content differs. The H atom content in the present study varies in the range of 0–0.50. The maximum atom number of a hydrogenated nanoparticle is ∼0.43 million. The time evolution of the atomic system is assumed to be within the framework of canonical (NVT) ensembles. Temperature is controlled at 300 K by a Nosé–Hoover thermostat.^21,22^ A velocity-based Verlet algorithm is adopted for the time integration together with a time step of 2.0 fs. After the construction of the nanoparticles, structural relaxation is first conducted in order to reach an energy favorable initial state. In what follows, nanoparticles are equilibrated dynamically at 300 K for about 1.0 ns when some H atoms jump between the octahedral and tetrahedral sites of the fcc lattice.^20^Fig. 1 depicts the hydrogenated Pd nanoparticles with different H atom contents after equilibration. It should be noted that hydrogenation leads to a remarkable volume change of the nanoparticles. To conduct uniaxial compression, two ideal planes were used as rigid planar indenters and a purely repulsive potential was adopted to describe the interactions between the rigid indenters and atoms in the nanoparticles, similar to the previous study.^3^ During compression, the rigid indenters move simultaneously towards the center of the nanoparticles at a speed of 0.1 Å ps^−1^.

The compression load is represented by the reactive force exerted on the indenters. The displacement of indenters is denoted as the compression depth. The ratio of the compression depth of indenters to the initial particle radius defines the compressive strain. In the simulation, a maximum strain of 0.4 is applied to each nanoparticle. To calculate the contact area, those atoms making contact with the indenters are first extracted, and the Delaunay triangulation algorithm is used to calculate the contact area. Dividing the compression load by the current contact area derives the average contact stress. The common neighbor analysis (CNA) algorithm is used to extract the atomic defects and their evolution inside the deformed nanoparticles.^23^ All the atomic configurations are visualized by the software OVITO,^24^ and an algorithm implemented in the software is used to extract dislocations and calculate the dislocation density.^25^

## 3. Results and discussions

### 3.1 Overall compression responses

**Fig. 2:**
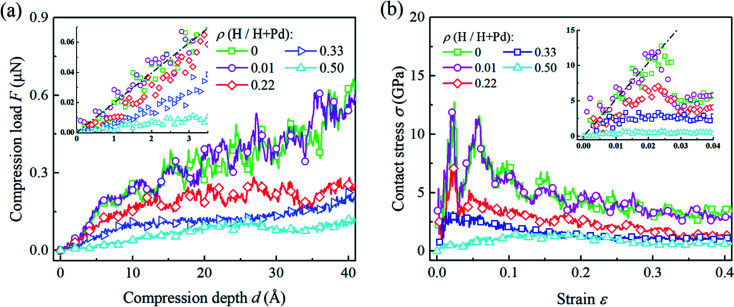
(a) The variation of compression load *versus* depth, and (b) the variation of the contact stress *versus* strain for Pd nanoparticles with different H atom contents. Dash lines shows the fitting of H-free nanoparticle based the flat punch contact model, eqn (2) and (3).

Fig. 2a shows several representative compression load-depth curves of the Pd nanoparticles with different H atom contents. It is seen that the compression load first increases linearly with the accumulation of the compression depth. The slope decreases with an increase in the content of H atom. In the following stage with further indentation loading, the linearity between load and depth vanished. For the cases having the H atom content lower than 0.3, apparent load fluctuation during the compressive process is displayed. In contrast, the variation of the compression load with respect to the depth is relatively smooth when the H atom content is higher than 0.3.

Fig. 2b depicts the variations of the average contact stress with respect to the compression strain. When the H atom content is lower than 0.3, the elastic and plastic stages are clearly distinguished from the stress–strain curves. As compression progresses, contact stress linearly increases up to a certain peak value, *i.e.*, the yield stress, followed by a prominent drop. The yielding points can be confirmed by checking the dislocation nucleation beneath the surface steps. When the H atom content is higher than 0.3, there is no sharp stress peak on the stress curve, and the average stress varies smoothly without apparent fluctuation. In this case, it is hard to determine the yielding points directly from the contact stress curves. Instead, the atomic scale deformation inside the nanoparticles should be examined. For the post-yielding stage, it is found that the contact stress level decreases with the increase in the H atom content. These dependencies of the overall responses on the H atom content are closely related to the deformation mechanism at the atomic scale, which will be discussed in detail in the following sections.

### 3.2 Elastic modulus and yield stress of the hydrogenated nanoparticles

Theoretical contact models have been successfully used to describe the elastic contact and extract the material properties of nanoparticles by uniaxial compression tests. Existing studies show that surface steps play important roles in the elastic contact of crystal nanoparticles. When the elastic contact is governed by the outmost surface step, the circular flat punch contact model is more precise to characterize the contact behavior.^3^ In the flat punch model, the compression load *F* exhibits a linear relationship with compression depth *δ*, which gives

2 *F* = 2E*aδ,

where E* is the reduced modulus along the compression orientation, and *a* is the contact radius. Furthermore, the variation of the average contact stress *p versus* compression strain *δ*/*R* is expressed as3
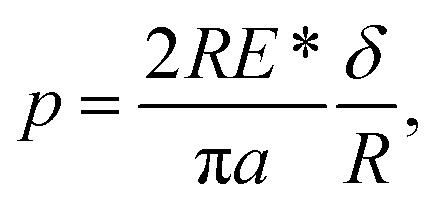
Eqn (2) and (3) can be used to extract the elastic modulus of nanoparticles based on the compression load-depth curves.

**Fig. 3:**
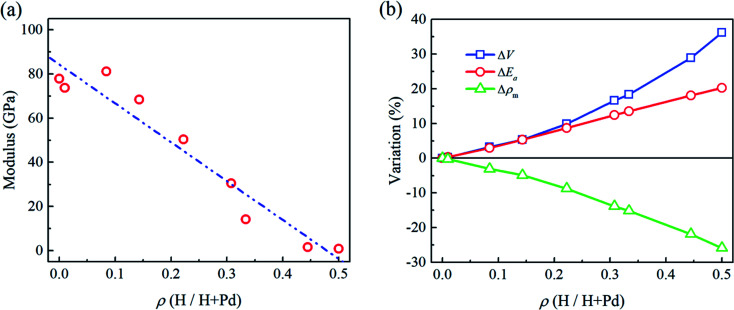
(a) Variation of the modulus of Pd nanoparticles *versus* the H atom content. Dash-dot-dot line is the linear fit of the data. (b) Variation of the particle volume *V*, the average cohesive energy per-atom *E*_a_, and the mass density *ρ*_m_ compared to those of the H-free nanoparticle *versus* the H atom content.

**Fig. 4:**
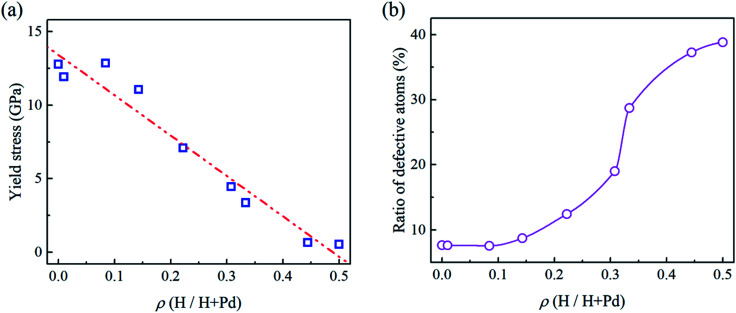
(a) Variation of the yielding stress of Pd nanoparticles *versus* the H atom content. Dash-dot-dot line is the linear fit of the data. (b) Ratio of the initial defective atoms inside nanoparticles *versus* the H atom content.

In Fig. 2a and b, the flat punch model shows good agreement with the simulation data in the linear elastic stage. Fig. 3a gives the extracted elastic modulus of the Pd nanoparticles with different H atom contents. The elastic modulus exhibits a linear dependence on the H atom content. The higher the H atom content, the lower the elastic modulus. The linear dependency of the decreasing elastic modulus on the H atom content has also been observed in the hydrogenated amorphous silicon nanoparticles,^26^ which is attributed to the initial defects and the decreased mass density induced by hydrogenation. In Fig. 3b, the variations of volume, density of mass, and the average cohesive energy per-atom for the Pd nanoparticles are calculated. It is shown that when the H atom content increases, the volume of the Pd nanoparticles could increase by 35% at maximum compared to the H-free nanoparticles, and the density decreases by 25%. Simultaneously, the average cohesive energy per-atom increases by 20%, indicating that the atomic lattice structure becomes unstable with higher H atom contents. These effects should be responsible for the decreasing modulus of the hydrogenated Pd nanoparticles. Besides, hydrogenation also has a tremendous influence on the yield stress of Pd nanoparticles. By examining the atomic deformation process, it is found that Pd nanoparticles with the H atom content in the range of 0–0.4 yield as long as the top-most surface step is flattened. For Pd nanoparticles having the H atom content in the range of 0.4–0.5, phase transition is the dominant deformation mode, and the yielding point is chosen as the moment when phase transition occurs in the contact region. Fig. 4a shows the variation in the yield stress with respect to the H atom content. Similar to the elastic modulus, the yield stress also shows a linear decreasing trend with the increase in the H atom content. Previous studies point out that surface steps have a great influence on the yield strength of crystal nanoparticles.^3,6,7^ For Pd nanoparticles, both the surface steps and the hydrogenation-induced initial defects influence the yield stress. To quantify the initial atomic defects prior to compression, Fig. 4b shows the fraction of defective atoms inside the Pd nanoparticles with respect to the H atom content. It is demonstrated that a higher H atom content induces a larger number of initial defects, and consequently, results in a lower yield stress.

### 3.3 Deformation mechanism at the atomic scale

In this section, the influences of hydrogenation on the deformation at the atomic scale are rendered in detail. Based on the atomic deformation characteristics, three types of deformation modes are revealed depending on the H atom content. When the H atom content is in the range of 0–0.3, dislocation nucleation and exhaustion dominate the plastic deformation. When the H atom content is in the range of 0.3–0.4, dislocation multiplication plays a key role in the plastic deformation. Moreover, the H atom content in the range of 0.4–0.5, phase transition is the dominant deformation mode. To demonstrate these three characteristic deformation modes, three Pd nanoparticles with H atom contents of 0.01, 0.33, and 0.50 are taken for instances.

**Fig. 5:**
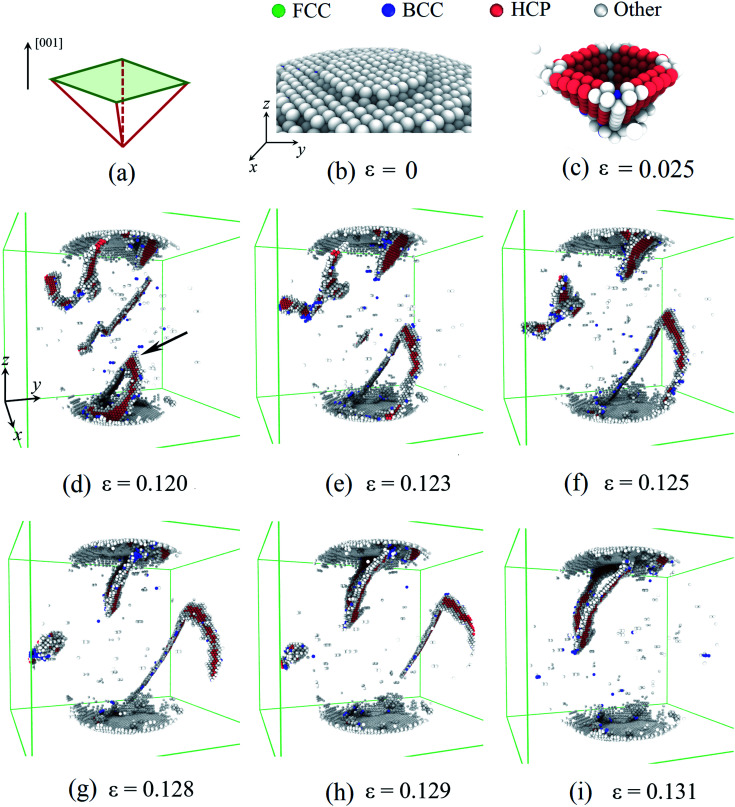
Plastic deformation of the Pd nanoparticle with the H atom content of 0.01: (a) the activated slip system under [001] compression. (b) The (001) surface step beneath the indenter. (c) The pyramid dislocation structure formed beneath the contact area when the nanoparticle yields. In the post-yielding deformation, a dislocation nucleates at the contact edge (marked by arrow) (d). After gliding across the whole nanoparticle (e–g), the dislocation finally reaches and annihilates at side free surface (h and i). In the figures, atoms are colored according to the CNA parameters. Atoms in fcc, bcc, hcp, and other structure are colored green, blue, red, and gray. In (d–i), atoms in the fcc lattice and at the initial surface are not shown for cleanness.

First, we considered the deformation of the Pd nanoparticles with the H atom content of 0.01. Fig. 5a and b show the activated slip system and initial surface steps under [001] compression. Because of the ultra-low H atom content, the influence of hydrogenation is negligible, and the yielding deformation is similar to the compression of other fcc crystal nanoparticles.^6,7^ The flat indenter first touches the top-most surface step and fully flattens the step when yielding occurs. The fringes of the surface step are preferable sites of dislocation nucleation because of stress concentration. Initial dislocations simultaneously nucleate on four equivalent slip systems, forming a pyramid dislocation hillock in the contact region (Fig. 5c), which has also been observed in the compression of other fcc crystal nanoparticles.^6,7^ In the post-yielding stage, full dislocations continue to nucleate at the contact edge as the accumulation of the compression depth (Fig. 5d). After nucleation, the jogged dislocation glides across the central region of the nanoparticles (Fig. 5e–g), and finally arrives and annihilates at the side free surface (Fig. 5h). Contrary to the copper nanoparticles,^7^ extended dislocations prevail in Pd nanoparticles with an ultra-low H atom content because of a much higher stacking fault energy of Pd.^27^ As dislocation could easily escape from the deformed nanoparticles (Fig. 5i), dislocation density is relatively low, leaving the nanoparticles starved of dislocation. When the compression advances, new dislocations have to continuously nucleate to sustain the deformation. The repetition of the dislocation exhaustion and nucleation causes fluctuation of the dislocation density in the post-yielding stage.

**Fig. 6:**
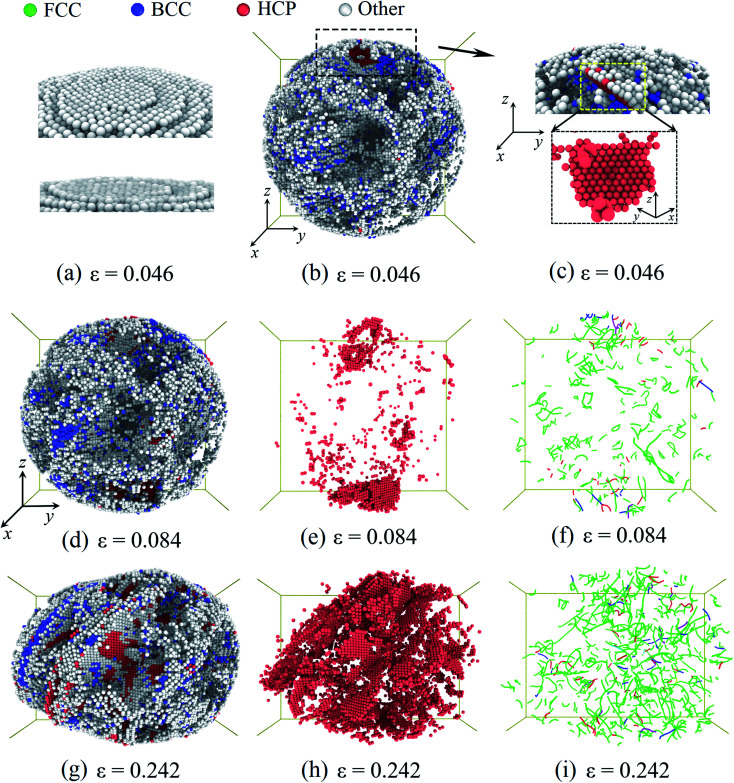
Plastic deformation of the Pd nanoparticles with the H content of 0.33: when particle yields at the strain of 0.046, (a) surface steps are partially flattened. (b) The distribution of the defective atoms inside the nanoparticles. (c) The nucleated stacking fault and the defective atoms in the contact region. At the strain of 0.084, the distribution of the defective atoms (d), the stacking faults (e), and dislocations (f). At the strain of 0.242, the distribution of the defective atoms (g), the stacking faults (h), and dislocations (i). The atom coloring scheme is same as that in Fig. 5. In (f) and (i), the colored lines represent the dislocation segments.

Then, we considered the deformation of the Pd nanoparticles with the H atom content of 0.33. From Fig. 4b, it is shown that the ratio of the initial defective atoms increases more than twice due to hydrogenation. Consequently, the plastic responses would be dramatically altered. Unlike the yielding of the nanoparticles with a lower H atom content range of 0–0.3, the top-most surface step is only partially flattened when the yielding occurs at the strain of 0.046 (Fig. 6a), and new stacking faults only form beneath the flattened fringe. Fig. 6b shows the distribution of the defective atoms in the nanoparticles. It is noted that beneath the contact surface the new stacking faults are located where there are fewer initial defective atoms (Fig. 6c). The pre-existing defective atoms under surface steps alter the local lattice structure, thus suppressing the nucleation of dislocations. Since the symmetric dislocation nucleation is broken-down, the pyramid dislocation hillocks no longer appear in the contact region. When compression advances, new dislocations and stacking faults first accumulate in the contact region (Fig. 6e and f). In the following stage, the multiplication of dislocations and stacking faults develop across the whole nanoparticle (Fig. 6h and i). In this case, plastic deformation is mainly sustained by dislocation multiplication. When the H atom content is low in the range of 0–0.3, the contact edges between the indenters and nanoparticle serve as the dislocation sources. The nucleated dislocation could glide through the center region without experiencing the heavy obstacles. In contrast, for nanoparticles with a higher H atom content in the range of 0.3–0.4, the pre-existing defects serve as both sources and obstacles to new dislocations, and the dislocation could nucleate at sites away from the contact region. The densely distributed defects and the entanglement of dislocations form a stable network, which prevents the dislocation from being exhausted at the free surface and contributes to the steady flow stress, as shown in Fig. 2b.

**Fig. 7:**
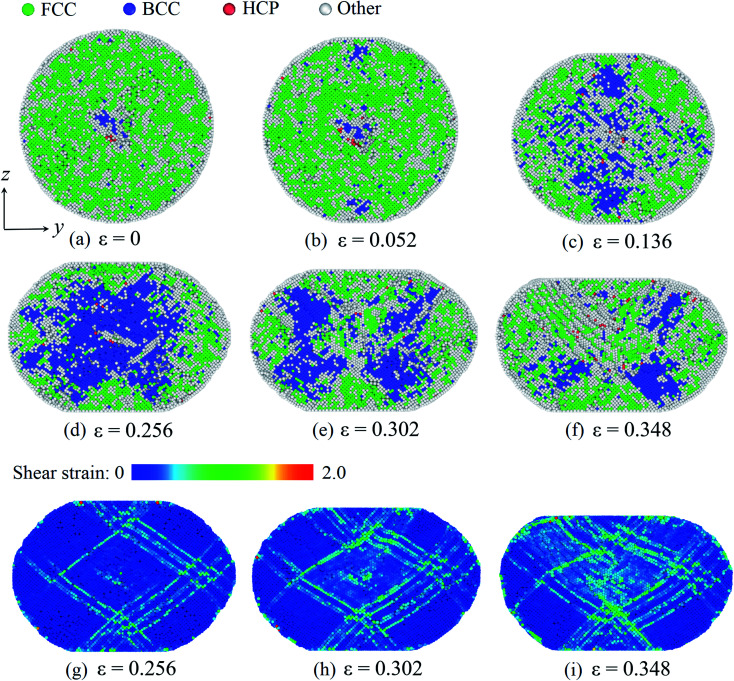
Phase transition of the Pd nanoparticles with the H content of 0.50: as deformation progresses, phase transition occurs in the contact region first (a and b) and then in the core region (c and d). With further deformation, phase transition partially recovers (e and f). The distribution of the atomic shear strain at different stages (g–i). In (a)–(d), atom coloring scheme is same as that in Fig. 5. In (g)–(i), atoms are colored according to the magnitude of the local shear strain.

At last, we consider the deformation of the Pd nanoparticle with the H atom content of 0.50. In this case, the nanoparticle has the highest ratio of defective atoms prior to compression, while the phase transition is the dominant deformation mode. Fig. 7a shows the cross section of the nanoparticle before compression. It is noted that the core region is rich in bcc atoms. When the yielding occurs, phase transition takes place in the contact regions as well, and the number of bcc atoms increases (Fig. 7b). As compression progresses, phase transition dominates the deformation, and massive atoms transmit into the bcc lattice (Fig. 7c and d). However, when the compression strain continues to increase (such as from 0.256 to 0.302 shown in Fig. 7e and f), the phase transition zone in the core region starts to decrease, and bcc atoms recover to fcc, hcp and other lattice structures. By examining the deformation, it is found that the atomic shear strain induces the recovery of the phase transition. Fig. 7g depicts the shear strain distribution when the phase transition zone is maximized. It is shown that the shear strain in the core region is relatively low, and the core region is also the primary phase transition zone. When compression strain increases further, shear deformation is gradually propagated to the core region, as revealed by the distribution of the shear strain (Fig. 7h and i). Consequently, the phase transition zone dwindles and partially recovers from the bcc lattice to other structure. When the H atom content is high, the nanoparticle experiences a larger volume expansion (Fig. 3b). At the beginning of compression, the nanoparticle is easily compressive. The dominant volume strain leads to the phase transition. When the deformation is large enough, shear deformation is propagated across the whole nanoparticle, which promotes the recovery of the phase transition.

**Fig. 8:**
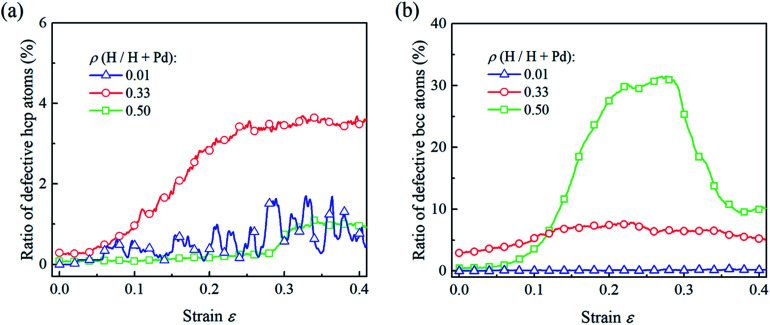
For the Pd nanoparticles having H atom contents of 0.01, 0.33, and 0.50, the ratios of the defective hcp atoms (a) and bcc atoms (b) vary with the compression strain.

To compare the deformation mechanisms of the Pd nanoparticles with H atom contents of 0.01, 0.33 and 0.50, the fractions of the defective atoms in hcp and bcc are calculated in Fig. 8. Atoms in hcp are primarily from the stacking generated during dislocation nucleation and gliding. In Fig. 8a, when the H atom content is 0.01, the nucleation of extended dislocations contributes to the increment of hcp atoms, and their exhaustion at surface leads to the decrease in hcp atoms. Thus, the fraction of defective hcp atoms fluctuates heavily with the nucleation and exhaustion of dislocations when strain is larger than 0.2. For the nanoparticles with the H atom content of 0.33, dislocation multiplication results in a stable dislocation network, and stacking faults are saturated with the multiplication of dislocations. Therefore, the fraction of hcp atoms gradually increases to a critical value and then remains constant. When the H atom content of the Pd nanoparticles is 0.50, phase transition is the dominant mechanism, the fraction of the hcp atom is at a lower level due to lack of dislocation multiplications. In Fig. 8b, for the Pd nanoparticles with the H atom content of 0.01 and 0.33, the dislocation nucleation does not contribute to the increase in bcc atoms and the fraction of bcc atoms in the nanoparticles does not increase dramatically. In the nanoparticle with the H atom content of 0.50, phase transition leads to the evident increment in the fraction of bcc atoms, and the following decreasing stems from the recovery of the phase transition driven by shear strain.

**Fig. 9:**
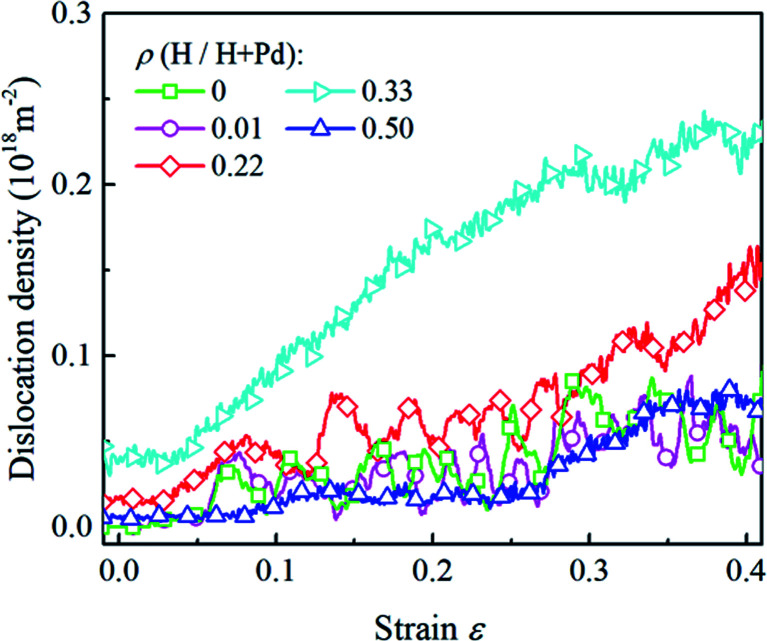
The variation in the dislocation densities in the Pd nanoparticles with different H atom contents *versus* the compressive strain.

The H atom content also influences the variation of the dislocation density, as shown in Fig. 9. It is noted that inside the nanoparticle with the lower H content in the range of 0–0.3, the dislocation density level is relatively low and fluctuates heavily owing to the repetition of dislocation nucleation and exhaustion at the free surface. When nanoparticle has the H atom content in the range of 0.3–0.4, the dislocation density keeps increasing in the whole stage without prominent fluctuation, indicating dislocation multiplication dominates the deformation. When the H atom content is in the range of 0.4–0.5, phase transition is a dominant mechanism, and the dislocation density is in a lower level without heavy fluctuation.

To address the influence of size effects, we have considered another two groups of nanoparticles with radii 5 and 15 nm. The hydrogenation-related phase transition still holds for different sized nanoparticles. However, the deformation mechanism as dislocation multiplication is less prominent in the smaller nanoparticles. The reason is that the smaller hydrogenated nanoparticles exhibit a larger surface-to-volume ratio and have a lower density of initial defects at the same level of the H atom content, which facilitate the dislocation escaping from the free surface, and lower the possibility of multiplication. Detailed influences of size effects on the hydrogenated nanoparticles will be studied in future study.

## 4. Conclusion

In the present study, molecular dynamics simulations are performed to investigate the influence of hydrogenation on the mechanical properties of Pd nanoparticles. The elastic modulus and yield stress of nanoparticles both decrease with the increment in the H atom content. Besides, the plastic responses are also significantly affected by the H atom content. When the H atom content is in the range of 0–0.3, nanoparticles yield *via* dislocation nucleation around the fringe of the top-most surface steps. Dislocation nucleation and annihilation at surface dominate the post-yielding stage, leading to the fluctuation of the dislocation density. If the H atom content increases in the range of 0.3–0.4, when the yielding occurs, dislocation nucleation around the surface steps is partially suppressed by the pre-existing defects in the contact region. In severe deformation, the densely distributed defects prevent dislocation from exhausting at surfaces, and dislocation multiplication mainly sustains the plastic deformation. When the H atom content is in the range of 0.4–0.5, the recoverable phase transition is the dominant deformation mechanism. The present study supplies a deformation landscape of the hydrogenated Pd nanoparticles, revealing the influence of the H atom content.

## Conflicts of interest

There are no conflicts to declare.

## Supplementary Material

RA-011-D0RA08974E-s001

RA-011-D0RA08974E-s002

RA-011-D0RA08974E-s003
