# Supplementary figures and images for: Influence of hydrogenation on the mechanical properties of Pd nanoparticles
Source: RSC Adv. 2021 Jan 13;11(5):3115–24. doi: 10.1039/d0ra08974e (PMC8693791; doi:10.1039/d0ra08974e)

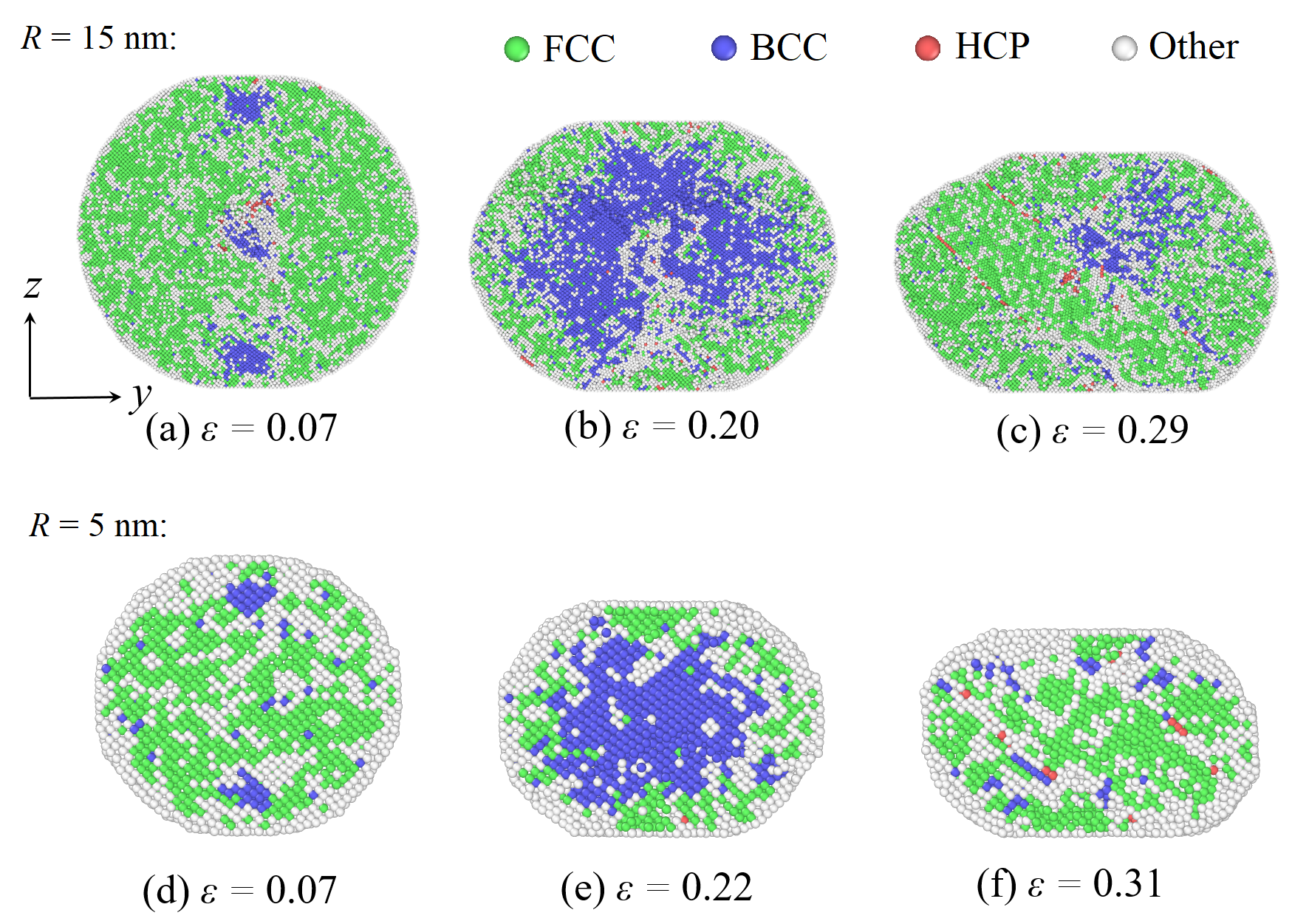

Supplement: RA-011-D0RA08974E-s002 [file RA-011-D0RA08974E-s002.tif]

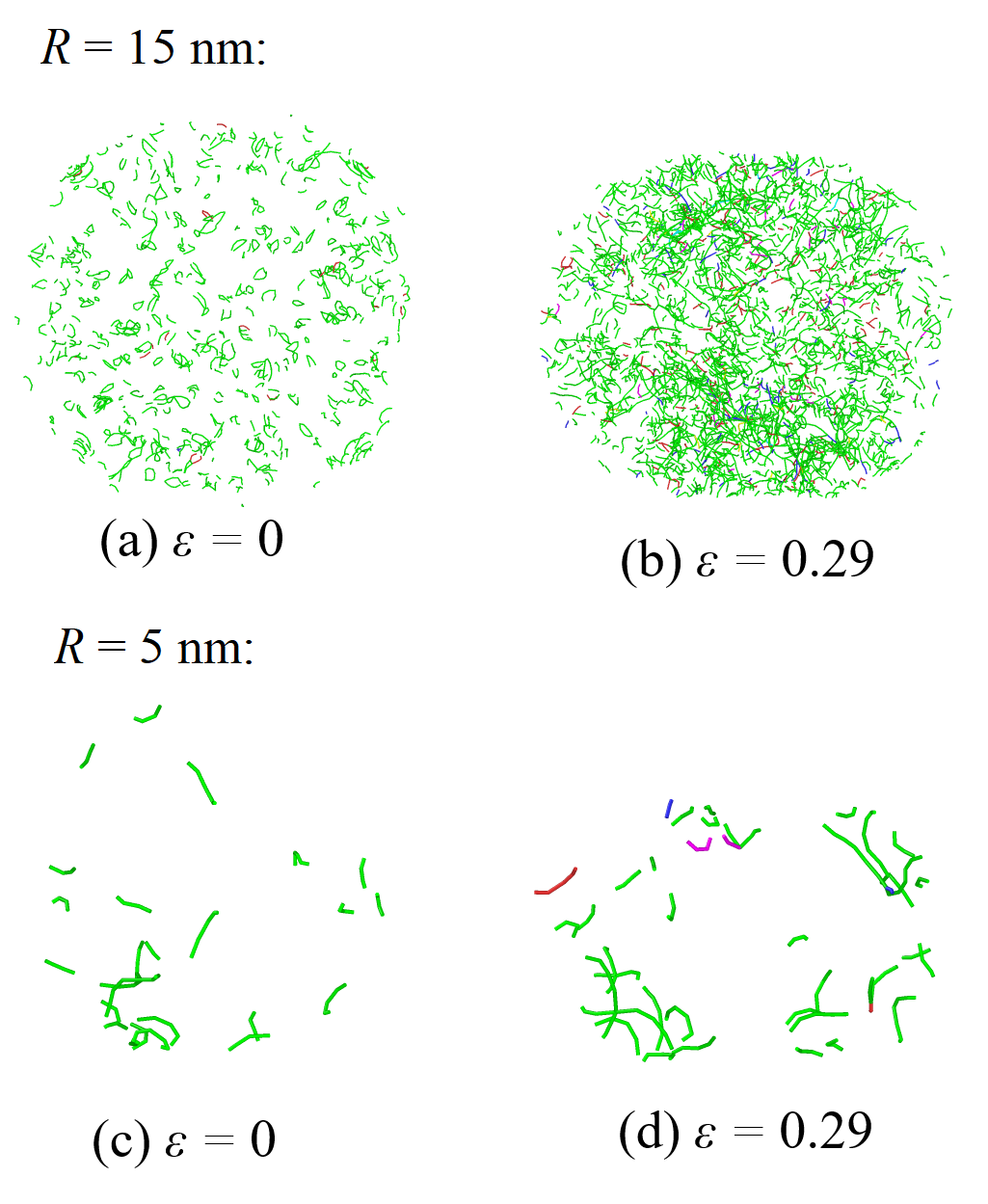

Supplement: RA-011-D0RA08974E-s003 [file RA-011-D0RA08974E-s003.tif]
